# Supplementary material for: A non‐canonical function of Plk4 in centriolar satellite integrity and ciliogenesis through PCM1 phosphorylation
Source: EMBO Rep. 2016 Jan 11;17(3):326–37. doi: 10.15252/embr.201541432 (PMC4772974; doi:10.15252/embr.201541432)
Supplement: Supplementary file 1 — Expanded View Figures PDF [file EMBR-17-326-s001.pdf]

Expanded View Figures

Figure EV1. Centriolar satellites become dispersed upon the depletion of Plk4, but not hSAS-6 in both U2OS and HeLa cells.

A, B U2OS cells were transfected with control, Plk4 or hSAS-6 siRNA. 48 h after transfection, cells were fixed and immunostained with antibodies against hMsd1 (A, green) or BBS4 (B, green) and anti-γ-tubulin antibodies (red). DNA was stained with DAPI (blue). Scale bars, 10 μm.

C Localisation of the dynein intermediate chain (DIC) is not altered upon Plk4 depletion. U2OS cells were treated with control or Plk4 siRNA and immunostained with antibodies against DIC (red) and Plk4 (green). DNA was stained with DAPI (blue). Scale bar, 5 μm.

D Efficient depletion of hSAS-6 and Plk4. Protein extracts were prepared from HeLa cells expressing centrin-GFP treated with hSAS-6 (left) or Plk4 siRNA (right). Immunoblotting was performed with the indicated antibodies. The position of protein size markers is shown on the right-hand side.

E, F HeLa cells expressing centrin-GFP were transfected with control, Plk4- or hSAS-6-silencing siRNA, fixed and stained with anti-PCM1 and anti-GFP antibodies or anti-hMsd1/SSX2IP and anti-GFP antibodies. Quantification of cells displaying the dispersed localisation of PCM1 (E) or hMsd1/SSX2IP (F) is shown. Data represent the mean + SD (> 200 cells, n = 3). Statistical analysis was performed using two-tailed unpaired Student's t-tests. **$P < 0.01$, ****$P < 0.0001$.

G Experimental scheme of G1 arrest and siRNA treatment. IF, immunofluorescence microscopy; WB, Western blotting (immunoblotting).

H, I G1 arrested U2OS cells were treated with control or Plk4 siRNA as in (G). Total cell extracts were prepared and immunoblotting was performed with the indicated antibodies (H). Band intensities of Plk4 in each sample were quantified using α-tubulin as a loading control (I). Data represent the mean + SD (> 200 cells, n = 3). Statistical analysis was performed using two-tailed unpaired Student's t-tests. *$P < 0.05$, **$P < 0.01$.

J Immunoblot showing the protein levels of ectopically expressed Plk4-myc constructs (WT, WT* and kinase dead KD*. *stands for siRNA resistant). U2OS cells were treated with control or Plk4 siRNA, followed by transfection of empty vector plasmids (EV) or those producing various Plk4 versions. Cell extracts were prepared and immunoblotting was performed with antibodies against myc and α-tubulin.

K U2OS cells were treated with control siRNA, and 48 h after transfection, plasmids producing siRNA-resistant Plk4-myc WT* or KD* were further introduced. 24 h after the second transfection, the cells were fixed and immunostained with PCM1 (green), myc (blue) and γ-tubulin antibodies (red). Cell peripheries are marked with solid lines, in which cells with yellow lines are successfully transfected (positive in myc signals), while those with white lines represent non-transfected cells. Scale bar, 5 μm.

© 2016 Cancer Research UK

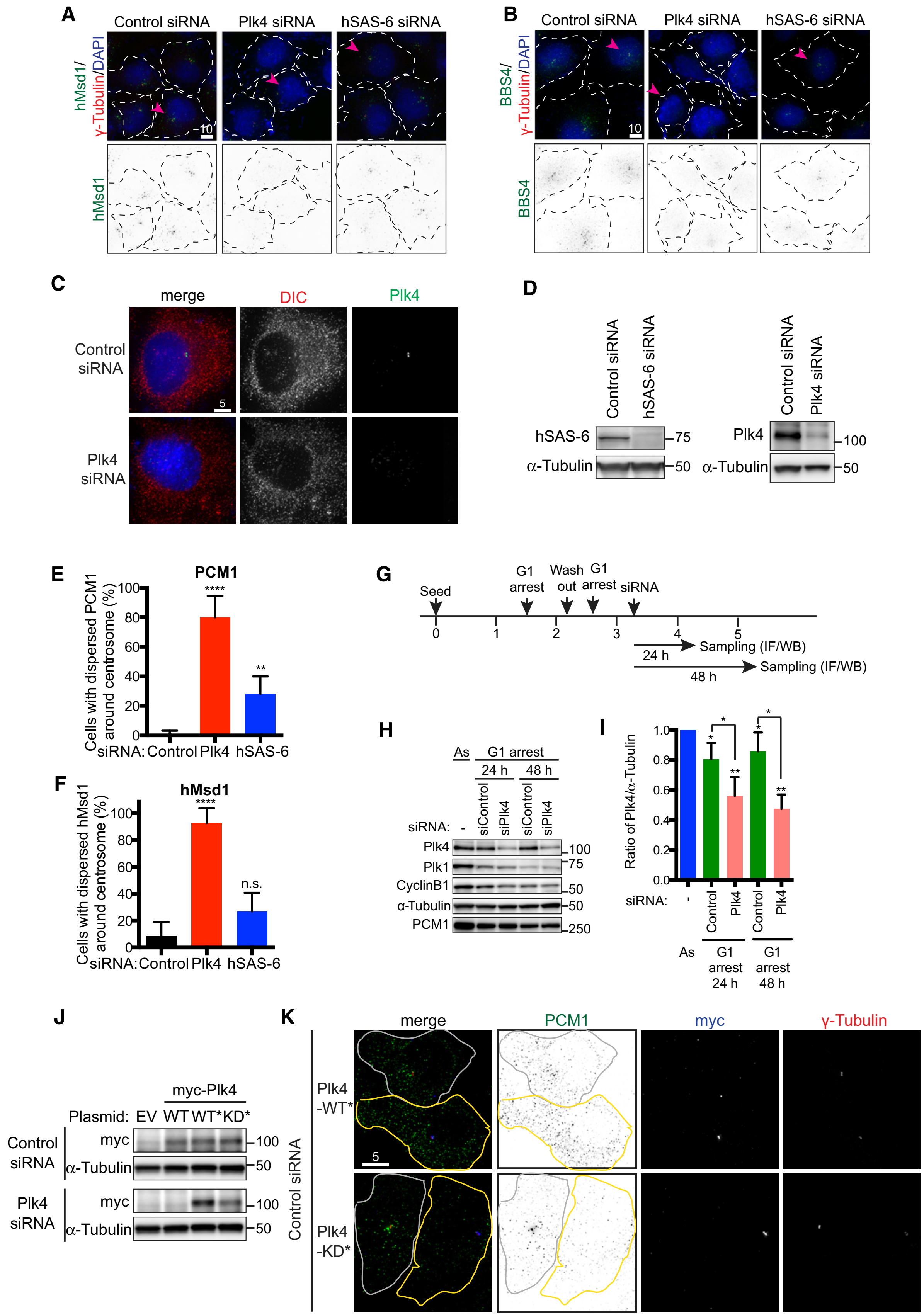

Figure EV1.

 © 2016 Cancer Research UK

Figure EV2. Identification of S372 within PCM1 as a phosphorylated residue dependent upon Plk4.

A Plk4-associated PCM1 is phosphorylated. U2OS cells were transfected with Plk4 siRNA, and empty vector plasmids (EV) or those producing siRNA-resistant wild-type or kinase-dead (KD) myc-Plk4 were introduced.

B Total cell extracts were prepared from HeLa cells treated with control or Plk4 siRNA, and immunoprecipitation was performed with an anti-PCM1 antibody. Patterns of Coomassie Brilliant Blue (CBB, top) staining and immunoblotting using an anti-PCM1 antibody (bottom) are shown. The position of a protein size marker is shown on the left-hand side.

C Table of identified phosphopeptides. Phosphorylated serines and the results of statistical evaluation are shown. Only S372 is hypophosphorylated in the samples prepared from Plk4 siRNA-treated cells.

D MS/MS sequence spectrum of S372-phosphorylated QAESLSLTR. -98 denotes the loss of H3PO4 and confirms the presence of phosphate. Although the identification is low scoring (MaxQuant score of 52), we have full-sequence coverage of the peptide including phosphorylation site position (localisation probability of 0.93).

E Semi-quantitative analysis of QAESLSLTR containing phosphorylated-S372 performed in Skyline. This phosphopeptide is present in lower abundance in the samples prepared from cells treated with Plk4 siRNA.

F siRNA-treated U2OS cells were transfected with empty vector plasmids (EV) or those producing various EGFP-connected PCM1 proteins (WT*, S372A*, S372D* or S372E*). Immunoblotting was performed by using the indicated antibodies.

G Validation of the S372 phospho-specific antibody. Samples prepared as in (F) were immunoprecipitated with an anti-GFP antibody and immunoblotted with S372 phospho-specific and anti-GFP antibodies. Asterisk shows non-specific bands. Phosphorylated bands were only detectable in immunoprecipitates, but not in the total extracts (input). Asterisk indicates a non-specific band.

© 2016 Cancer Research UK

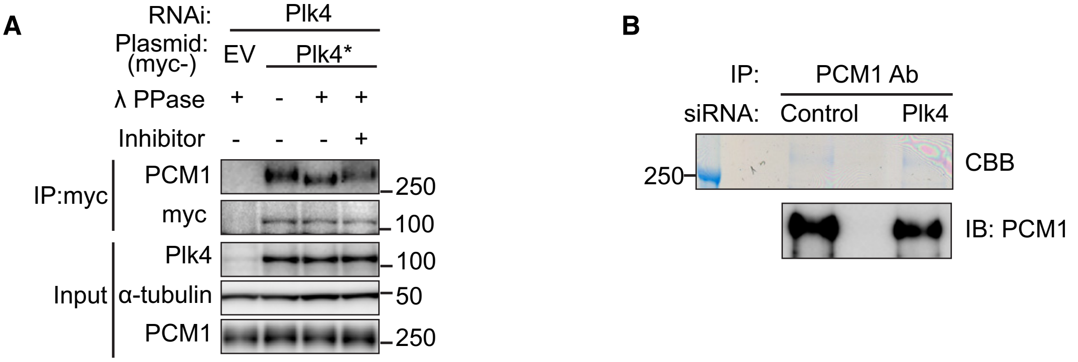

C

Localisation probability	PEP	Score	Position	Modified sequence	Phospho (STY) Probabilities	Number of Phospho (STY)
0.92964	0.0188477	52	372	_QAESLS(ph)LTR_	SQAES(0.032)LS(0.93)LT(0.039)R372	1
0.5	0.0202383	41	643	_S(ph)SLVDEHPEDAEFEQK_	S(0.5)S(0.5)LVDEHPEDAEFEQK	1
0.5	0.0202383	41	644	_S(ph)SLVDEHPEDAEFEQK_	S(0.5)S(0.5)LVDEHPEDAEFEQK	1
0.997945	2.37E-06	78	861	_RQGLAETAS(ph)PVAVSLR_	RQGLAET(0.002)AS(0.998)PVAVSLR	1
	2.37E-06	78	861	_QGLAETAS(ph)PVAVSLR_		1
0.985736	0.00395539	74	1231	_TGFSVS(ph)VEK_	TGFS(0.014)VS(0.986)VEK	1
0.999671	0.00308065	43	1722	_ILEDHGS(ph)PAGEIDDEDKDKDETETVK_	ILEDHGS(1)PAGEIDDEDKDKDETETVK	1

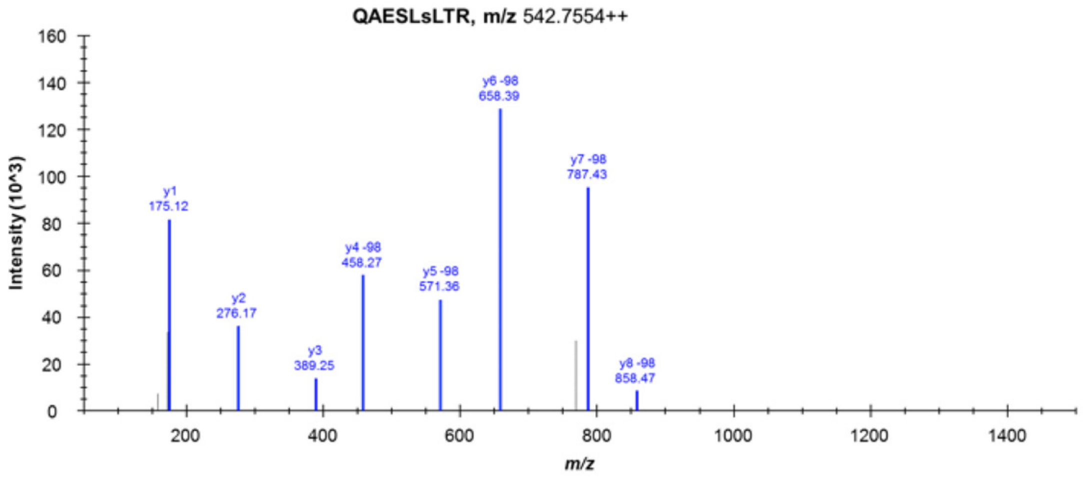

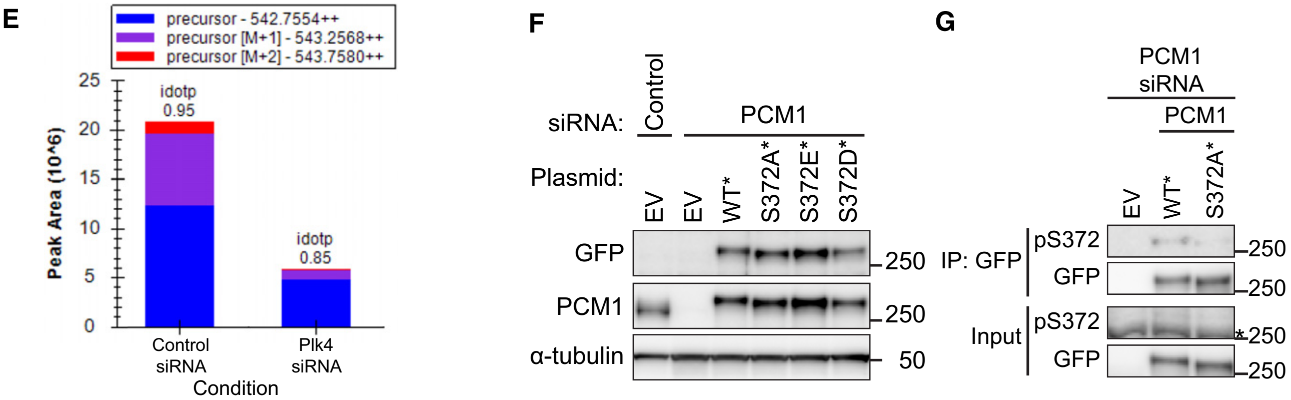

Figure EV2.

 © 2016 Cancer Research UK

Figure EV3. PCM1-S327A* and PCM1-S327E* result in the dispersion and compromised motility of centriolar satellite, respectively.

A–C PCM1 siRNA-treated U2OS cells were transfected with plasmids producing EGFP-PCM1-WT* or -S372A*. Immunofluorescence microscopy was performed with antibodies against γ-tubulin (arrowheads, red), GFP (blue, PCM1) and hMsd1/SSX2IP (green, A), BBS4 (green, B) or Cep290 (green, C). Quantification data are shown on the bottom of each panel. Data represent the mean + SD (> 200 cells, n = 3). Statistical analysis was performed using two-tailed unpaired Student's t-tests. *P < 0.05, **P < 0.01, ***P < 0.001. Scale bar, 5 μm.

D Kymograph analysis of PCM1-WT* and PCM1-S372E* particles. U2OS cells treated with PCM1 siRNA were transfected with plasmids producing EGFP-PCM1-WT* or -S372E*, and time-lapse imaging was performed (n = 20 for PCM1-WT*; n = 19 for PCM1-S372E*). Scale bar, 1 μm.

E–G Quantification of PCM1 trajectory shown in (D). The average velocity (E), maximal velocity (F) and length (G) of PCM1 particles are measured for cells producing PCM1-WT* or PCM1-S372E*. Data represent the mean + SD (> 200 cells, n = 3). Statistical analysis was performed using two-tailed unpaired Student's t-tests. ****P < 0.0001.

H U2OS cells were treated with PCM1 siRNA and further transfected with plasmids producing EGFP-PCM1-S372E*. Immunofluorescence microscopy was performed with antibodies against γ-tubulin (red, centrosome marker), GFP (blue) and hMsd1/SSX2IP (green, top), BBS4 (green, middle) or Cep290 (green, bottom). Enlarged images around the centrosomal region are shown in insets (in which colocalising signals of EGFP-PCM1-S327E* and satellite components are marked by arrowheads). Scale bars, 1 μm (inset), 5 μm.

© 2016 Cancer Research UK

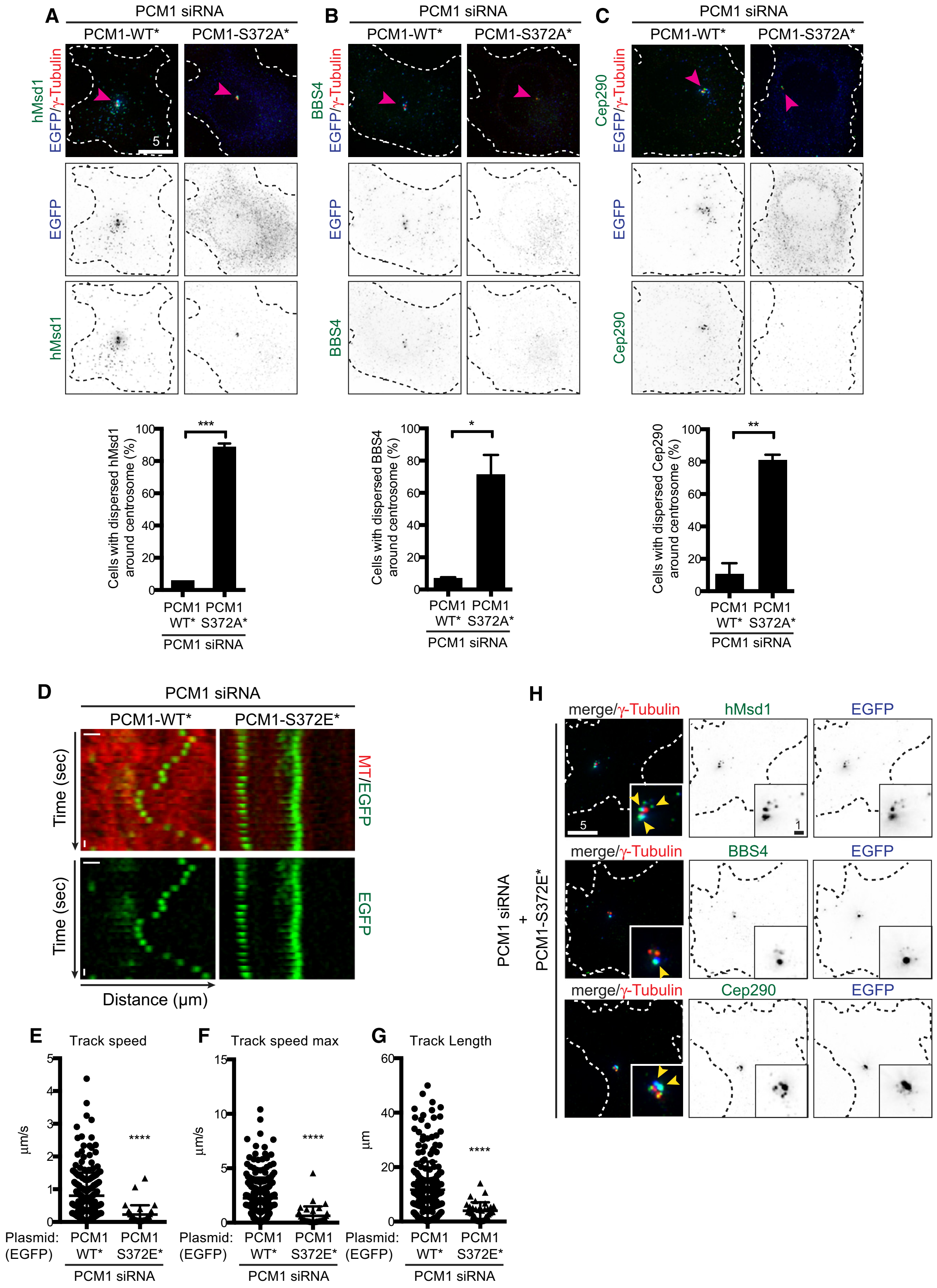

Figure EV3.

 © 2016 Cancer Research UK

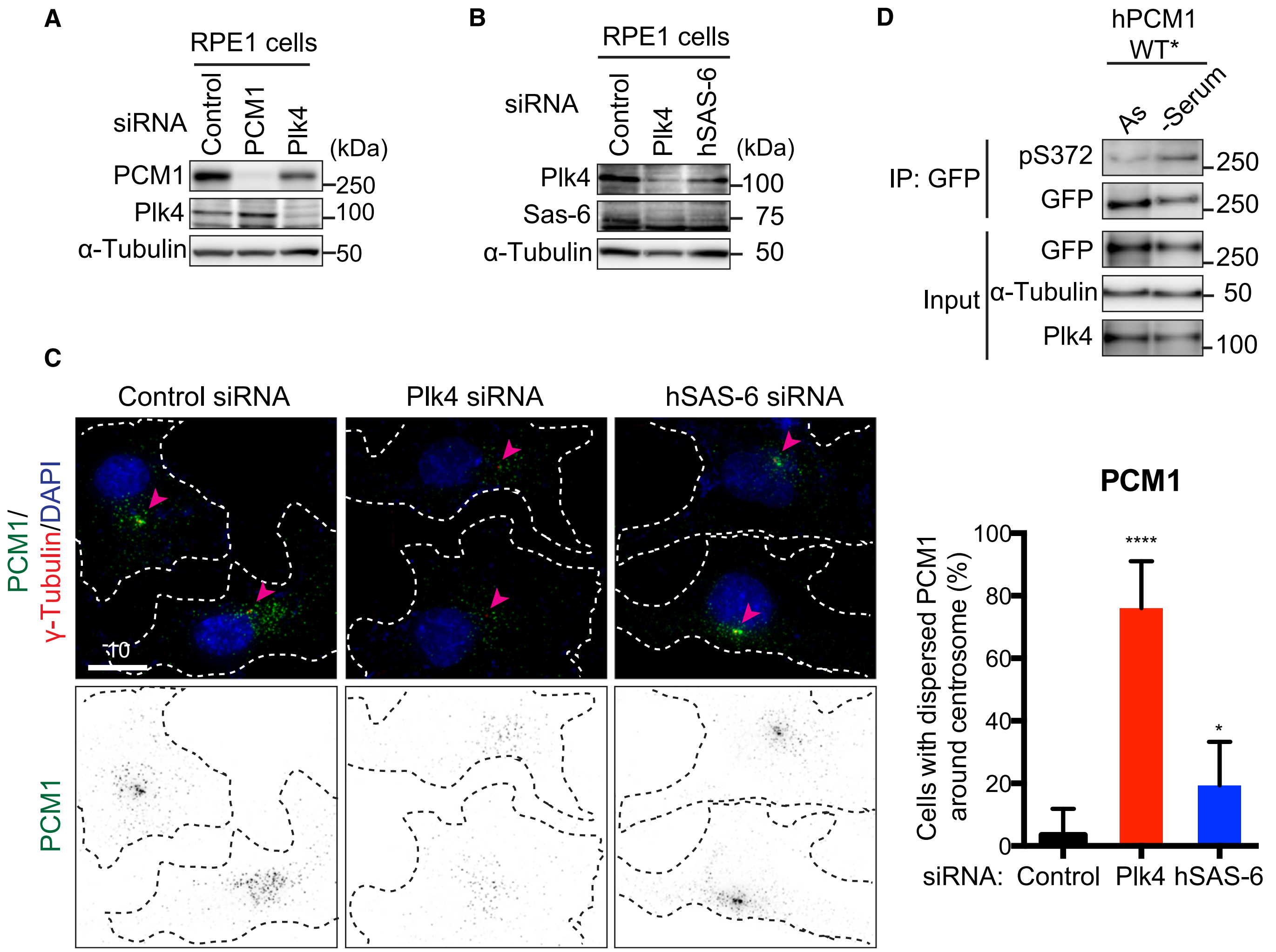

Figure EV4. Plk4, but not hSAS-6, depletion leads to the dispersion of PCM1 from the centrosomal region in RPE-1 cells.

A, B hTERT-RPE-1 cells were treated with control, PCM1 or Plk4 siRNA. Cell extracts were prepared and immunoblotting was performed with the indicated antibodies. The position of protein size markers is shown on the right-hand side.

C Plk4, but not hSAS-6, depletion leads to the dispersal of PCM1 from the pericentrosomal region. hTERT-RPE-1 cells prepared as in (A, B) were immunostained with anti-PCM1 (green) and anti-γ-tubulin antibodies (red). DNA was stained with DAPI (blue). The centrosomes are marked with arrowheads, and cell peripheries are shown with doted lines. Quantification of cells displaying dispersed PCM1 signals is shown on the right. Data represent the mean + SD (> 150 cells, n = 3). Statistical analysis (right) was performed using two-tailed unpaired Student's t-tests. *$P < 0.05$, ****$P < 0.0001$. Scale bar, 10 μm.

D hTERT-RPE-1 cells were transfected with plasmids producing EGFP-PCM1-WT* and asynchronously cultured or serum-starved to induce ciliogenesis for 48 h. Protein extracts were prepared and immunoprecipitation performed with an anti-GFP antibody, followed by immunoblotting with the indicated antibodies.

Source data are available online for this figure.

© 2016 Cancer Research UK
