# Supplementary figures and images for: A non‐canonical function of Plk4 in centriolar satellite integrity and ciliogenesis through PCM1 phosphorylation
Source: EMBO Rep. 2016 Jan 11;17(3):326–37. doi: 10.15252/embr.201541432 (PMC4772974; doi:10.15252/embr.201541432)

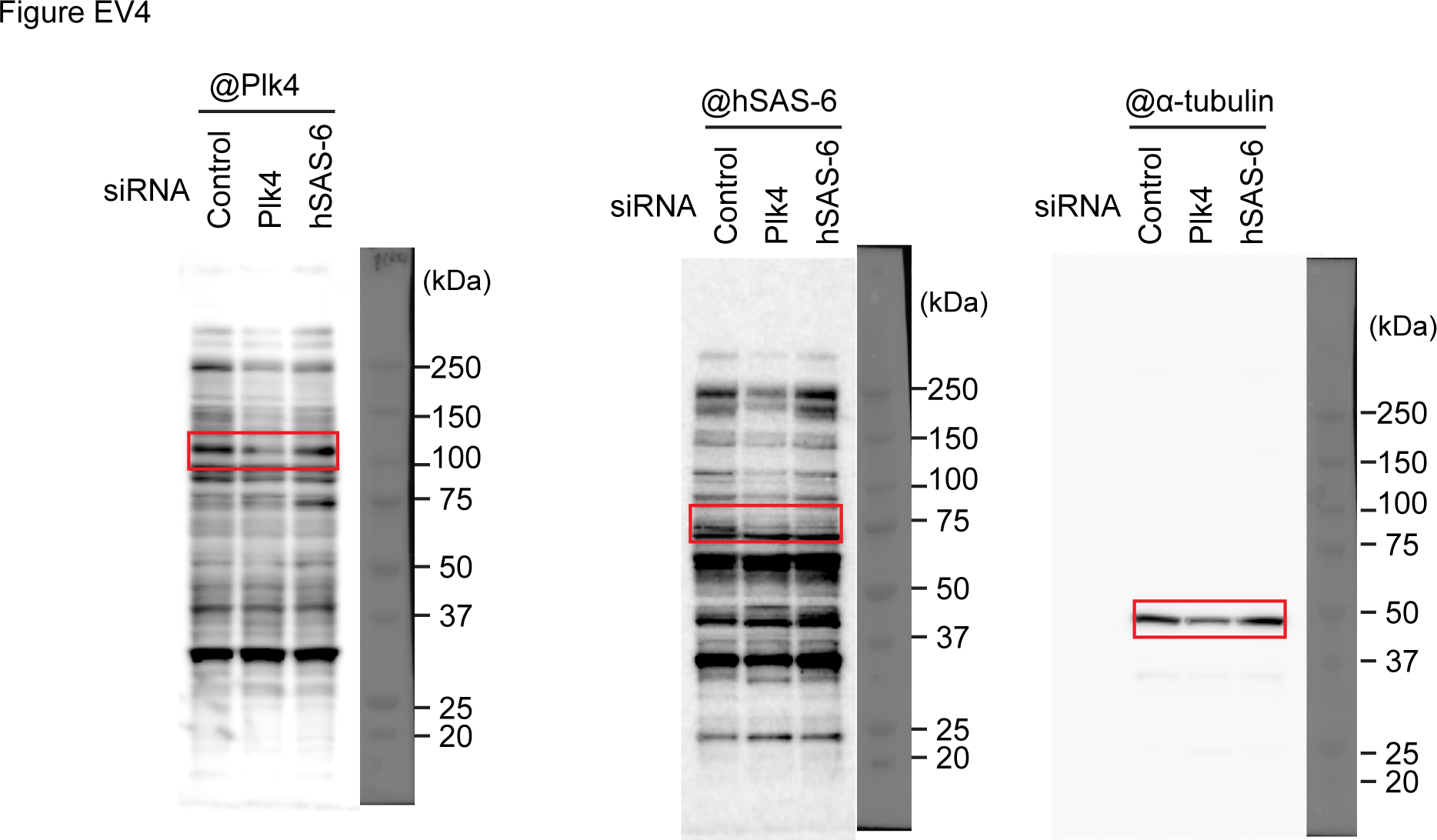

Supplement: Supplementary file 2 — Source Data for Expanded View [file EMBR-17-326-s002.zip › EV_source_data/Figure EV4 Source Data.tif]

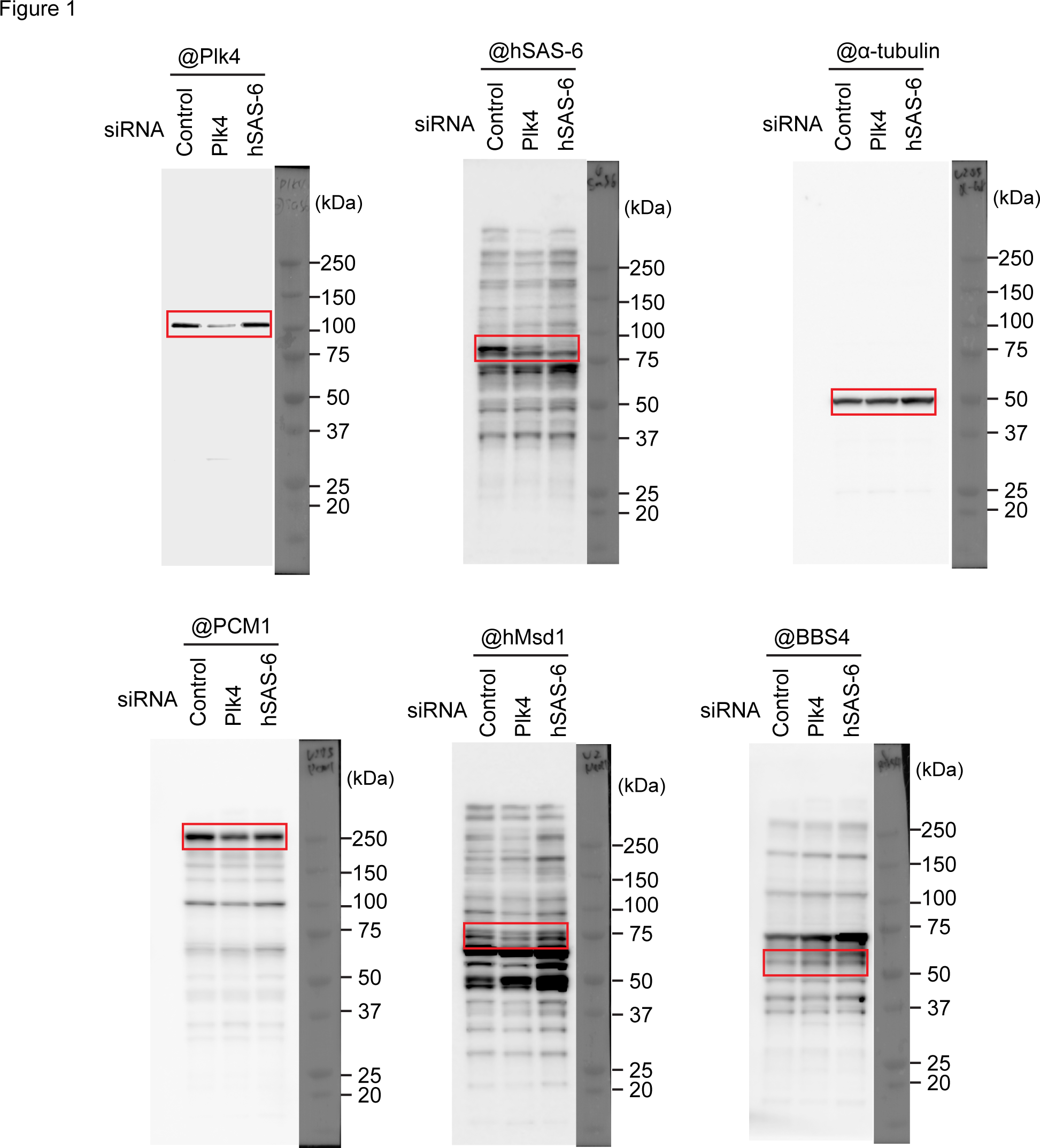

Supplement: Supplementary file 4 — Source Data for Figure 1 [file EMBR-17-326-s003.tif]
